# *Manilkara zapota* (L.) P. Royen Leaf Mitigates Colitis-Associated Colon Cancer through Anti-inflammatory Modulation in BALB/C Mice

**DOI:** 10.1155/2024/1137696

**Published:** 2024-09-10

**Authors:** Norain Mohd Tamsir, Norhaizan Mohd Esa, Nurul Husna Shafie, Hazilawati Hamzah

**Affiliations:** ^1^ Department of Nutrition Faculty of Medicine and Health Sciences Universiti Putra Malaysia 43400, Serdang, Selangor, Malaysia; ^2^ Natural Medicine and Product Research Laboratory (NaturMeds) Institute of Bioscience Universiti Putra Malaysia 43400, Serdang, Selangor, Malaysia; ^3^ Laboratory of UPM-MAKNA Cancer Research Institute of Bioscience Universiti Putra Malaysia 43400, Serdang, Selangor, Malaysia; ^4^ Department of Veterinary Pathology and Microbiology Faculty of Veterinary Medicine Universiti Putra Malaysia 43400, Serdang, Selangor, Malaysia

## Abstract

Colitis-associated colon cancer (CAC) arises from prolonged inflammation of the inner colon lining. An alternative approach to treating or preventing CAC involves the use of natural products such as *Manilkara zapota* (L.) P. Royen or *M. zapota,* which has been studied for its medicinal and pharmacological properties. Previous research has demonstrated the anticancer effects of *M. zapota* leaf aqueous extract (MZLAE) on colon cancer cells. However, no animal study has investigated the effects of MZLAE on CAC. Therefore, this study aimed to assess the potential anti-inflammatory effects of MZLAE on CAC in mice. In the present study, CAC was induced using azoxymethane (AOM) and dextran sodium sulphate (DSS). The mice were randomly assigned into five groups: (a) normal, (b) AOM/DSS, (c) AOM/DSS + 50 mg/kg MZLAE, (d) AOM/DSS + 100 mg/kg MZLAE, and (e) AOM/DSS + 200 mg/kg MZLAE. Various parameters including disease activity index (DAI), colon length and weight, reactive oxygen species (ROS), superoxide, superoxide dismutase (SOD), histopathological assessment, and proinflammatory cytokines expression were analysed. The results indicated that MZLAE improved DAI scores, colon length, colon histological dysplasia and inflammation scores, and SOD level, while also reducing ROS production and expression of proinflammatory cytokines (tumour necrosis factor-alpha (TNF- *α*) and interleukin 6 (IL-6)). In conclusion, this study suggests that MZLAE may serve as a promising source of antioxidants and anti-inflammatory agents for alleviating CAC.

## 1. Introduction

Colorectal cancer (CRC) is the third most common cancer and the second leading cause of cancer-related death worldwide [1]. Although CRC rates have traditionally been higher in Western nations compared to Asia, the incidence of CRC is surprisingly rising in Asian countries. In Malaysia, CRC is the most prevalent cancer among men and the second most common cancer among women [2]. Alarmingly, there is an increasing number of CRC cases among younger individuals [3]. Prolonged ulcerative colitis (UC), a type of inflammatory bowel disease (IBD), is a recognised risk factor for CRC. UC causes inflammation of the colon mucosa and, when CRC develops in people with UC, it is known as colitis-associated colorectal cancer (CAC). The risk of developing CRC is significantly higher by twofold in UC patients compared to the general population. CAC is generally more aggressive than spontaneous or sporadic CRC, leading to a poorer prognosis and higher mortality risk [4]. Although sporadic CRC usually arises from raised adenomatous polyps, the dysplasia that precedes CAC often occurs in flat lesions that are invisible endoscopically. The timing and order of the changes in carcinogenesis distinguish CAC from spontaneous CRC.

The pathogenesis and mechanisms underlying IBD-CRC remain largely unknown. One theory posits that persistent inflammation accelerates the mutation process, resulting in cells that appear normal, but have the potential to become cancerous. In contrast, the “Big Bang” theory suggests that cancerous cells with multiple mutations arise suddenly [5]. Inflammation is recognised as the main driver of neoplastic changes and progression, which plays a crucial role in dysplasia, and is the most significant factor in the development of CRC [6].

Inflammation is pivotal in all stages of tumour development, from initiation to metastasis. According to Fantini and Guadagni, chronic inflammation contributes to carcinogenesis through two primary mechanisms: a direct mechanism involving oxidative stress and damage to deoxyribonucleic acid (DNA) and indirect mechanisms mediated by cytokines produced by inflammatory cells and intestinal epithelial cells in response to inflammation. Furthermore, inflammation induces intestinal dysbiosis, which contributes to CAC by generating reactive metabolites and carcinogens and disrupting the epithelial barrier. This disruption leads to increased intestinal permeability, linked to ongoing stimulation of the mucosal immune system and chronic inflammation [7]. Therefore, the treatment of inflammation in the colon is considered crucial in the prevention of CAC.

Two key molecular pathways involved are the nuclear factor kappa-light-chain enhancer of activator B cells (NF-*κβ*) and signal transducer and activator of transcription 3 (STAT3) [8]. Chronic inflammation leads to damage to DNA induced by oxidative stress, which can activate oncogenes and inactivate tumour suppressor genes. Consequently, markers of oxidative damage and DNA double-strand break gradually and accumulate in the inflammation-dysplasia-carcinoma sequence, rather than the adenoma-carcinoma sequence typical of spontaneous CRC [9]. Due to the importance of controlling inflammation in the colon, biologic treatments and immunomodulators that target inflammatory pathways are considered essential for treating IBD and preventing its progression to CRC [10].

At present, CAC is typically managed similarly to UC treatment, utilising nonsteroidal anti-inflammatory drugs (NSAIDs) like 5-aminosalicylic acid (5-ASA). However, these medications often cause adverse effects such as diarrhoea and nephritis [11]. Antibodies, such as antitumour necrosis factor (anti-TNF), are also used to treat CAC, known for their safety and efficacy, although they are expensive and require injection administration [12]. Alongside pharmacological interventions, surgical procedures are also considered for CAC treatment. Nonetheless, prior research discovered that surgical management of CAC necessitates more extensive medical attention and carries an elevated risk of complications [12]. Consequently, the search for a noninvasive therapy or prevention measure for CAC, devoid of negative impacts, remains imperative.

*M. zapota*, commonly known as sapodilla, or “chikoo,” has a rich history of use in traditional medicine. Indigenous to southern Mexico and Central and South America, this plant is also cultivated in various Asian countries including India, the Philippines, Malaysia, Vietnam, and Thailand [13]. Its leaves are renowned for their diverse pharmacological and medicinal properties such as antioxidant [14], anti-inflammatory [15], antidiarrhoeal [16], antidiabetic [17], antihypercholesterolemic [18], and antityrosinase [19] activities, among others. Previous *in vitro* studies highlighted the anticancer effects of MZLAE on hepatocellular carcinoma (HepG2) [20] and colon cancer (HT29) cells [21] by modulating the transcription expression of the extracellular signal-regulated kinase 1/protein kinase B/C-Jun N-terminal protein kinase 1 (ERK1/Akt1/JNK1) and integration site/beta-catenin (Wnt/*β*-catenin) signalling pathways, respectively. However, to date, the anticancer properties of MZLAE in CAC *in vivo* have not been documented. Therefore, the current study aimed to investigate the impact of MZLAE on CAC mice.

## 2. Materials and Methods

### 2.1. *Manilkara zapota* (L.) P. Royen Leaf Aqueous Extract

The leaves of *M. zapota* were harvested and submitted for species verification at the Herbarium, Institute of Biosciences, Universiti Putra Malaysia (UPM), where the voucher number is MFI 0036/19. After collection, the leaves were thoroughly washed and dried in a ventilated dryer at 35°C until they reached an appropriate dryness. Subsequently, the dried leaves were finely ground to prepare them for extraction. The extraction process followed the method described by Wan Nurhayati et al., with some adjustments [22] In brief, the dried leaf powder was soaked in 1 : 10 ratio (w/v) of distilled water and agitated in an orbital shaker at 100 rpm at room temperature for 24 hours. The filtrate was then filtered and freeze-dried to obtain a concentrated powder.

### 2.2. Animal Study

**Figure 1:**
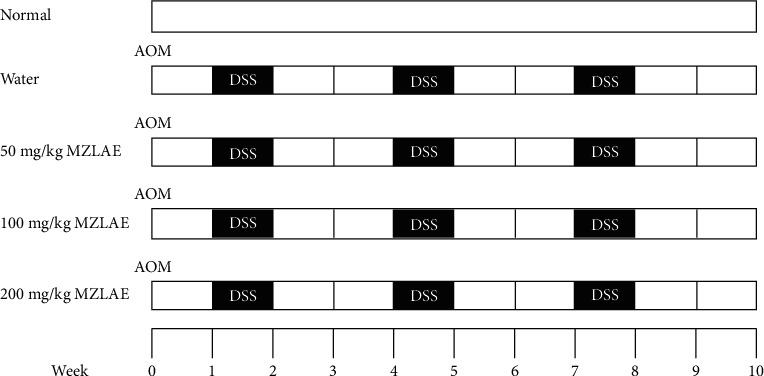
Schematic overview of the experimental timeline.

Forty female BALB/c mice, aged 6–8 weeks with an average body weight (bw) of 18−20 g, were acquired from the Animal Resource unit at the Faculty of Veterinary Medicine, UPM, Serdang, Selangor. The study's procedures involving animal care were ethically approved by the Institutional Animal Care and Use Committee (IACUC) in June 2016, under reference number UPM/IACUC/AUP-R030/2016. The mice were housed at the animal house within the Faculty of Medicine and Health Sciences, UPM, Serdang, Selangor. They underwent a two-week acclimatisation period, during which they were provided with unrestricted access to standard mouse pellets and plain drinking water. The animals were maintained under standard laboratory conditions, which included a 12-hour light/dark cycle, a temperature of 22 ± 2°C, and a relative humidity of 30–70% [23] After acclimatisation, the mice were randomly assigned to five groups as illustrated in Figure 1. MZLAE was administered orally via gavage daily.

On day 1 of the experiment, mice in groups 2, 3, 4, and 5 received a single intraperitoneal injection (i. p) of 10 mg/kg of bw AOM (Sigma-Aldrich, MO, USA). Subsequently, during the following week, mice in the same groups were subjected to the first cycle of 2% (w/v) DSS (MP Biomedicals, CA, USA) dissolved in their drinking water for one week. This was followed by a two-week recovery period during which the mice received normal drinking water. DSS administration was repeated in three cycles. Throughout the experiment, mice were closely monitored for any clinical signs or abnormalities resulting from the induction of CAC using AOM/DSS, as well as MZLAE administration. Food consumption was monitored, and individual body weights were recorded weekly until week 10. At the end of week 10, mice were euthanised following a fasting period of 3-4 hours. Blood and organs were collected for further analysis.

### 2.3. Evaluation of the Disease Activity Index (DAI)

The DAI is a commonly used tool to assess colitis symptoms [24]. Throughout the experimental duration, general clinical indicators such as body weight loss, stool consistency, and the presence of blood in the stool were monitored and recorded daily. The DAI scoring criteria were determined by summing the scores and then dividing the total by three (Table 1).

### 2.4. Serum and Organ Collection

At week 10, all mice were sacrificed by exsanguination via cardiac puncture under fully sedation using intraperitoneal injection of a mixture containing ketamine and xylazine. The blood samples were placed in a plain tube, then stored on ice before separation. The serum was separated by centrifuging the blood at 3000 rpm for 15 minutes at 4°C [23]. The colon was harvested, washed with normal saline, and weighed to determine organ coefficients, calculated as the organ-to-body weight ratio (mg/g). Colons were then fixed in 10% neutral-buffered formalin for further analysis. Tissues were sectioned, embedded in paraffin, and sliced to a thickness of 4–6 *µ*m, followed by staining with hematoxylin and eosin (H&E) stain for histopathological evaluation. The histological dysplasia score was determined by assessing the severity of colon tissue damage, with a score of 0 indicating the absence of dysplasia, 2 indicating low-grade dysplasia, and 4 indicating high-grade dysplasia. The dysplastic index was calculated as the average score [25]. In addition, colon inflammation severity was evaluated using the method described by Nowak et al. [26]. Inflammation severity was graded on a scale from 0 to 3 (0: no inflammation, 1: mild, 2: moderate, and 3: severe), and the thickness of the inflammatory involvement ranged from 0 to 3 (0: no inflammation, 1: mucosa, 2: mucosa plus submucosa, and 3: transmural). Epithelial damage severity was scored from 0 to 3 (0: intact epithelium, 1: disruption of architectural structure, 2: erosion, and 3: ulceration), and the extent of the lesions was categorised as 0 (no lesion), 1 (punctuate), 2 (multifocal), and 3 (diffuse).

### 2.5. Colon Homogenisation

In brief, 5 mg of the colon was homogenised in ice-cold phosphate-buffered saline (PBS) or radioimmunoprecipitation buffer (RIPA) along with 3 *µ*L of protease inhibitor cocktail (Merck Millipore, MA, USA). The homogenate was then incubated on ice for 20 min, followed by centrifugation at 10,000 rpm at 4°C for 30 min [27]. The resulting supernatant was collected for further analysis. The protein concentration of the supernatant was quantified using the Pierce TM bicinchoninic acid (BCA) protein assay kit (Thermo Scientific, IL, USA) following the manufacturer's instructions with bovine serum albumin (BSA) as a standard and expressed as a mg/mL sample.

### 2.6. Measurement of Intracellular Reactive Oxygen Species (ROS) Generation and Superoxide Production

The cell level of ROS generation was determined using a 2′, 7′ diacetate fluorescent probe (DCFH-DA) (Nacalai Tesque Inc., Kyoto, Japan) following the protocol described by Zizzo et al. with some modifications [28]. The colon supernatant was incubated with 2.5 mM DCFH in ethanol for 1 hour at 37°C. The fluorescent DCF resulting from the ROS oxidation reaction was measured fluorometrically at an excitation wavelength of 485 nm and an emission wavelength of 520 nm. ROS production was measured as a relative fluorescence unit.

The production in the colon was determined using dihydroethidium (DHE) (Cayman Chemical, MI, USA). In brief, the colon supernatant was incubated in 3.3 mM DHE at 37°C for 20 minutes. The fluorescence intensity was measured with an excitation wavelength of 530 nm and an emission wavelength of 620 nm. The intensity of DHE alone was recorded to establish a zero point, and the superoxide values were expressed as the intensity per milligram of protein. The superoxide value of the control group was standardized to 100% [29].

### 2.7. Measurement of Superoxide Dismutase (SOD)

Superoxide dismutase activity in serum was determined using a Mouse Superoxide Dismutase 1 (SOD1) soluble (competitive) ELISA kit following the manufacturer's instructions (Elabscience, Wuhan, China).

### 2.8. Protein Expression by Western Blot Analysis

Western blot analysis was performed following the method described by Nagendraprabhu and Sudhandiran with slight modifications [30]. The protein extracts were heated in a boiling water bath for 5 min, then subjected to 12.5% sodium dodecyl sulphate-polyacrylamide gel electrophoresis and transferred to polyvinylidene difluoride membranes using a transfer apparatus (Bio-Rad Laboratories Inc., USA). The membranes were blocked overnight at 4°C with a blocking reagent, bovine serum albumin (BSA) in Tris-Tween buffered saline (TBST). Subsequently, the membranes were incubated with primary antibodies against TNF-*α*, IL-6 (Santa Cruz Biotechnology Inc., CA, USA), and beta-actin (*β*-actin) (Solarbio Life Sciences, Beijing, China) at the appropriate dilutions recommended by the supplier overnight at 4°C. Subsequently, the membranes were incubated with the corresponding horseradish peroxidase-conjugated secondary antibody (Santa Cruz Biotechnology, Santa Cruz, CA, USA) for 1 h. Protein-antibody complexes were visualised using Clarity Western ECL substrate (Bio-Rad Laboratories Inc., USA) and quantification was performed using ImageJ software (National Institutes of Health, Bethesda, MD, USA).

### 2.9. Statistical Analysis

The data obtained were subjected to statistical analysis using the Statistical Package for Social Sciences software (IBM SPSS version 23.0, Chicago, IL, USA). The results were presented as mean or median ± standard error of the mean (SEM). For normally distributed data, a one-way analysis of variance (ANOVA) was employed to detect any significant differences between groups, followed by Tukey's post hoc test to identify specific groups with significant differences. Non-normally distributed data were analysed using the nonparametric Kruskal–Wallis H test. The significance threshold was established at *p*  <  0.05 for the tests.

## 3. Results

### 3.1. MZLAE Ameliorated DAI Scores of AOM/DSS-Induced CAC Mice

**Figure 2:**
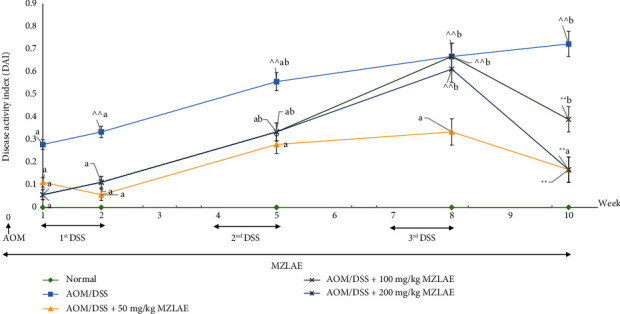
DAI score of CAC mice (*n* = 6). ^a^Values with different letters on the same line (group) indicate significant differences between weeks at *p* <  0.05. ^∗∗^ indicates a significant difference compared to the AOM/DSS group (*p* < 0.05). ^  ^ indicates a significant difference compared to the normal group (*p* < 0.05). Week 1: AOM administration; week 2: first DSS cycle; week 5: second DSS cycle; week 8: third DSS cycle; week 10: end of the study. AOM: azoxymethane; DSS: dextran sodium sulphate; MZLAE: *Manilkara zapota* leaf aqueous extract.

To investigate the effect of MZLAE on DAI scores, parameters such as body weight loss, stool consistency, and blood in stools were evaluated.  Figure 2 illustrates the DAI scores of MZLAE-treated mice from week 1 to week 10. Following administration of AOM and DSS, several mice exhibited stool inconsistency, bloody stools, and weight loss. At the end of week ten, the AOM/DSS group showed elevated DAI scores compared to the normal group. However, CAC groups treated with MZLAE exhibited significantly lower DAI scores compared to the AOM/DSS group, indicating the potential of MZLAE to improve DAI scores. The DAI score for CAC mice treated with 100 and 200 mg/kg MZLAE was significantly higher than in the normal group (*p* < 0.05). This result was consistent with a previous study, in which CAC mice treated with extra virgin olive oil also exhibited higher DAI after multiple cycles of DSS administration [31]. The severity and mortality of CAC can vary significantly due to its complex pathophysiology, leading to nonlinear responses to treatment. In some cases, higher doses of treatment may trigger immune responses or other physiological reactions that negate the beneficial effects observed at lower doses [32]. In contrast, the DAI score for CAC mice treated with 50 mg/kg MZLAE was not significantly different from the normal group (*p* > 0.05) suggesting that this lower dose did not worsen the condition and might be closer to the baseline health status of normal mice.

### 3.2. MZLAE Improved Colon Length and Weight in CAC Mice

Table 2 highlights a notable disparity in colon length among the experimental groups. The untreated AOM/DSS group demonstrated significantly shorter colon length compared to the normal group (*p* < 0.05). In contrast, CAC mice supplemented with 50 mg/kg, 100 mg/kg, and 200 mg/kg MZLAE exhibited significantly longer colon lengths than the untreated AOM/DSS group (*p* < 0.05).

Colon weights were also assessed after the sacrifice of the mice at week 10. The study revealed that the highest colon weight-length ratio was shown by the AOM/DSS group, whereas the AOM/DSS + 200 mg/kg MZLAE group exhibited the lowest ratio. However, no significant differences were observed in the weight-length ratio between the treatment groups (*p* > 0.05).

### 3.3. MZLAE Attenuates Colon Histological Dysplasia and Inflammation of AOM/DSS-Induced Mice

**Figure 3:**
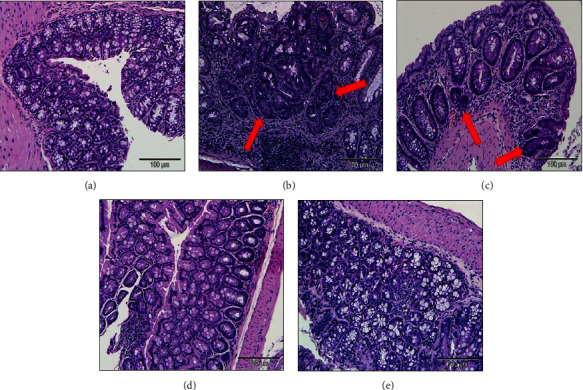
Histological evaluation of mouse colonic dysplasia using H&E staining **( ***n* = 3): (a) normal group, (b) AOM/DSS group (arrow shows distorted crypts in high-grade dysplasia), (c) AOM/DSS + 50 mg/kg MZLAE group (arrow shows low-grade dysplasia), (d) AOM/DSS + 100 mg/kg MZLAE, and (e) AOM/DSS + 200 mg/kg MZLAE group (100 *µ*m = magnification or 20X). AOM: azoxymethane; DSS: dextran sodium sulphate; MZLAE: *Manilkara zapota* leaf aqueous extract.

Figure 3 shows the morphological evaluation of dysplasia in the colons of mice in the five experimental groups. The images illustrate histological alterations in the crypt structure of colonic cells in AOM/DSS mice, classified as low-grade dysplasia (LGD) or high-grade dysplasia (HGD), in contrast to the colons of mice in the normal group. Subsequently, histological dysplasia was quantified and scored, as described in Table 3.

Data indicate that colons of normal mice exhibited a normal architecture without signs of dysplasia, resulting in a score of 0 (no dysplasia). In contrast, colons from mice induced by AOM/DSS showed low-grade dysplasia (LGD) at a significantly higher rate compared to the normal group (*p*  <  0.05). However, the number of colonic dysplasia was significantly reduced in the AOM/DSS + 200 mg/kg group compared to the AOM/DSS group (*p*  <  0.05).

Furthermore, mice colons exhibited high-grade dysplasia (HGD) at a markedly higher rate compared to the normal group (*p* < 0.05). Although the number of dysplasia decreased after MZLAE treatment, the reduction was not significant compared to the AOM/DSS group (*p*  >  0.05).

**Figure 4:**
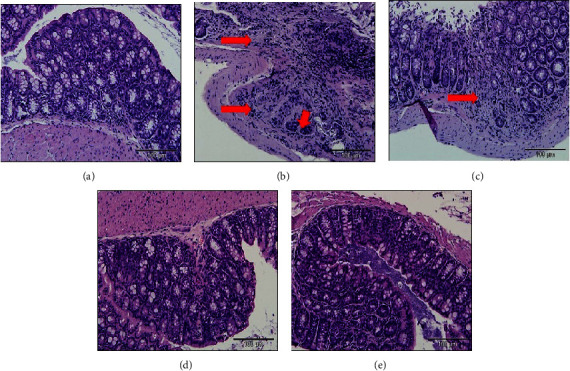
Histological evaluation of colonic inflammation in CAC mice (*n* = 3)**:** (a) normal, (b) AOM/DSS (long arrow shows infiltration of inflammatory cells and short arrow shows destruction of the mucosal cell architecture), (c) AOM/DSS + 50 mg/kg MZLAE (arrow shows inflammation), (d) AOM/DSS + 100 mg/kg MZLAE, and (e) AOM/DSS + 200 mg/kg MZLAE (100 *µ*m = magnification or 20X). AOM: azoxymethane; DSS: dextran sodium sulphate; MZLAE: *Manilkara zapota* leaf aqueous extract.

Figure 4 illustrates the histological profile of colon mucosal inflammation in experimental mice. It highlights the infiltration of inflammatory cells and crypt destruction observed in CAC-induced mice, which were absent in the normal group.

Histological assessment was conducted to evaluate the severity, thickness, and extent of colonic damage as summarised in Table 4. No signs of inflammation or damage were observed in the colons of normal mice resulting in a score of 0. In comparison, the inflammation score was significantly elevated in the AOM/DSS group compared to the normal group (*p* < 0.05). However, all treatment groups showed a notable reduction in inflammation scores compared to the AOM/DSS group (*p* < 0.05). The thickness of the inflammatory involvement scores in the AOM/DSS group was significantly higher compared to the normal group. Notably, the AOM/DSS + 200 mg/kg MZLAE group demonstrated a significant improvement in the thickness of the inflammatory involvement score compared to the AOM/DSS group (*p* < 0.05). Similarly, the severity of epithelial damage scores in the AOM/DSS group was significantly higher compared to the normal group (*p* < 0.05). Treatment with MZLAE resulted in significant reductions in the severity of epithelial damage compared to the untreated counterparts (*p* < 0.05). The extent of the lesion score of the AOM/DSS group was significantly higher than that of the normal group (*p* < 0.05). However, treatment with MZLAE led to a significant improvement in the extent of lesion scores compared to the AOM/DSS group (*p* < 0.05). Thus, MZLAE-treated mice demonstrated enhanced colon histological scoring, indicating improvement in inflammatory involvement thickness, epithelial damage severity, and lesion extent in a dose-dependent manner.

### 3.4. MZLAE Reduced Intracellular Reactive Oxygen Species (ROS) in AOM/DSS-Induced Mice

**Figure 5:**
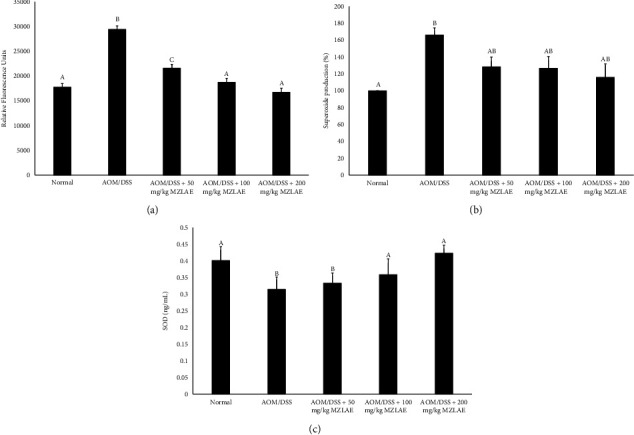
Effect of MZLAE on ROS production (a), superoxide production (b), and SOD level of CAC mice (c) (*n* = 3). ^a^Values with different letters indicate significant differences at *p* < 0.05. AOM: azoxymethane; DSS: dextran sodium sulphate; MZLAE: *Manilkara zapota* leaf aqueous extract; SOD: superoxide dismutase.

Furthermore, to evaluate the effects of MZLAE on ROS in CAC mice, intracellular ROS production was determined. As shown in Figure 5(a), the AOM/DSS group exhibited elevated ROS production compared to the normal group (*p* < 0.05). However, treatment with MZLAE significantly decreased ROS levels in all CAC groups (*p* < 0.05).

### 3.5. Colon Superoxide Production

The results presented in Figure 5(b) clearly show a higher superoxide production in the AOM/DSS group compared to the normal group (*p*  < 0.05). Interestingly, MZLAE supplementation at all concentrations exhibited a trend toward reducing superoxide formation, although the difference was not statistically significant.

### 3.6. Superoxide Dismutase (SOD) Activity

Figure 5(c) illustrates that the SOD levels in mice from the AOM/DSS group were significantly lower compared to the normal group (*p* < 0.05). Remarkably, significant increases in SOD levels were observed in the CAC group receiving 100 mg/kg MZLAE and 200 mg/kg MZLAE compared to the untreated AOM/DSS group (*p* < 0.05).

### 3.7. MZLAE Suppressed the Expression of Proinflammatory Cytokines in AOM/DSS-Induced Mice

**Figure 6:**
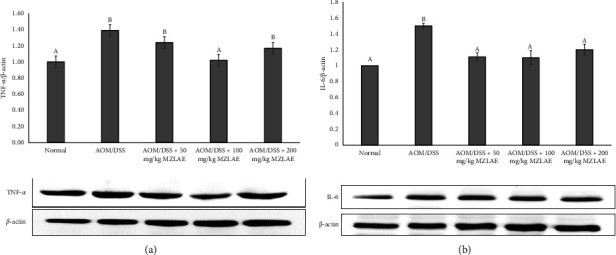
Effect of MZLAE on the expression of (a) TNF-*α* and (b) IL-6 by Western blot (*n* = 3). ^a^Values with different letters indicate significant differences at *p* < 0.05. AOM: azoxymethane; DSS: dextran sodium sulphate; MZLAE: *Manilkara zapota* leaf aqueous extract.

To further explore the impact of MZLAE on CAC, the expression of TNF-*α* and IL-6 cytokines in mouse colon tissues was analysed. In Figure 6(a), it is evident that TNF-*α* expression was significantly increased in the AOM/DSS group compared to the normal group (*p* < 0.05). However, a significant reduction in TNF-*α* expression was observed in the AOM/DSS + 100 mg/kg MZLAE compared to the AOM/DSS group (*p* < 0.05).

Similarly, Figure 6(b) demonstrates a significant increase in IL-6 protein expression in the AOM/DSS group compared to the normal group (*p* < 0.05). Interestingly, IL-6 levels were significantly reduced after MZLAE supplementation at all concentrations (*p* < 0.05). These findings suggest that MZLAE supplementation leads to a decrease in IL-6 levels, which consequently reduces ROS, superoxide, inflammation, and diarrhoea in CAC mice.

## 4. Discussions

In the search for alternatives to anti-inflammatory drugs, recent research has focused on natural products known for their minimal toxicity and limited adverse effects on cells or organs. This study explores the potential of the evergreen *Manilkara zapota* (*M. zapota*), which has a long history of medicinal and pharmacological use. Previous research has highlighted the remarkable antioxidant capacities of MZLAE compared to other parts of the plant [14]. In addition, *in vitro* studies have demonstrated the nontoxic nature and beneficial properties of MZLAE [20, 21]. However, there is a lack of data on the *in vivo* effects of MZLAE, particularly in the context of colitis-associated colon cancer. Therefore, investigating the anti-inflammatory effects of MZLAE *in vivo* holds significant promise and relevance.

In the field of biomedical research, rodents are the primary species utilised for experimental studies. Specifically, for the chemically induced CAC model, BALB/c mice are frequently used due to their increased susceptibility to chemical induction [33]. In this study, DSS and AOM were used for CAC induction, which closely mimics human CAC. DSS, a synthetic-sulphated polysaccharide, is known for its high toxicity and its ability to alter the integrity of the intestinal epithelial barrier [34]. Furthermore, the presence of oxide in AOM is believed to be responsible for inducing carcinomas of the colon. Previous research has indicated that the reduction in body weight observed after administration of AOM/DSS serves as a marker of inflammation [35]. This weight loss may be attributed to the damaging effects of DSS on the colonic epithelium, triggering an inflammatory response and subsequent colitis.

Snider et al. have reported that the AOM/DSS model produces a pathology manifested by severe colitis, characterised by a significant loss of body weight and bloody diarrhoea [36]. To assess the severity of the disease, the researchers used the disease activity index (DAI) which considers parameters such as weight loss, stool consistency, and presence of blood in stool throughout the study. Mice induced with AOM/DSS exhibited higher DAI scores due to weight loss, diarrhoea, and/or blood in the stool. However, treatment with MZLAE resulted in improved DAI scores, likely attributed to its known anti-inflammatory [15, 16, 37] and antidiarrhoeal [16, 38] effects. Similar findings were observed in studies in which CAC mice received leaf extract of *Bryophyllum pinnatum* (Lamarck) [39] and black lentil and its isolated compound [40], which significantly reduced DAI scores following DSS administration.

Chronic inflammation in the colon can lead to anatomic abnormalities such as strictures of inflammatory polyps (commonly known as “psuedopolyps”) and ultimately shortening of the colon [5] Colon shortening is recognised as a hallmark of colitis in mice, and previous studies indicate that it may be an indicator of inflammation severity [41, 42]. Furthermore, an increase in the weight-to-length ratio of the colon suggests mucosal thickening [43]. In this study, mice treated with MZLAE exhibited an improved colon length, indicating the anti-inflammatory effects of MZLAE.

Furthermore, histopathological analysis of tissue samples also provides supportive evidence for the disease condition [44]. Colonic histopathological evaluation is considered more precise than other methods for measuring inflammatory markers such as plasma C-reactive protein and albumin. This is because histopathological evaluation allows the detection of the site of inflammation in affected areas, unlike biomarkers detected in blood, which may not pinpoint the exact location of inflammation [45]. One of the morphological parameters observed in neoplastic precursor lesions is dysplasia. Dysplasia is identified by structural alterations and changes in the nuclear and cytoplasmic characteristics of the colon, which can affect the progression toward colon cancer. The extent of dysplasia is evaluated by examining the interplay of abnormalities in nuclear morphology, cytoplasmic characteristics, and architectural arrangement within the crypt epithelium [46]. Repeated administration of DSS can lead to chronic colitis, colorectal dysplasia, and eventually cancers.

To complement the histopathological evaluation, other indicators of inflammatory-related conditions, such as ROS levels, were measured. ROS perform diverse functions in normal physiological processes within cells, where a specific level of ROS is essential to maintain regular cellular function. However, an imbalance in redox homeostasis can occur, leading to ROS accumulation and subsequently oxidative stress. This imbalance has been implicated in various human diseases, including but not limited to IBD and colon cancer [47]. ROS levels are elevated in CRC cells compared to normal colonic cells. This increase in ROS levels can alter the inflammatory response in the intestine, leading to intestinal mucosal barrier damage, modification of lipids and proteins, and DNA damage [48]. These effects highlight the importance of assessing ROS levels as a marker of inflammation and oxidative stress in conditions such as IBD and colon cancer.

Among the various types of ROS, superoxide is particularly detrimental to cells and is a well-known proinflammatory factor implicated in conditions such as UC. Furthermore, the levels of superoxide dismutase (SOD), an antioxidant enzyme, were also analysed. SOD plays a crucial role in cellular defense by catalysing the dismutation of superoxide radicals, generated during oxygen metabolism, into molecular oxygen (O_2_) and hydrogen peroxide (H_2_O_2_). A decrease in SOD activity has been observed in cancer cells, since it serves to protect cells from excessive oxygen radicals, ageing, and other harmful substances [49]. Previous studies have demonstrated that MZLAE reduces lipid peroxidation by reducing malondialdehyde (MDA) levels and increasing enzyme antioxidant (catalase) levels in HT-29 colorectal cancer cells [20]. These findings further support the notion that MZLAE exhibits robust antioxidant activity both *in vitro* and *in vivo*.

**Figure 7:**
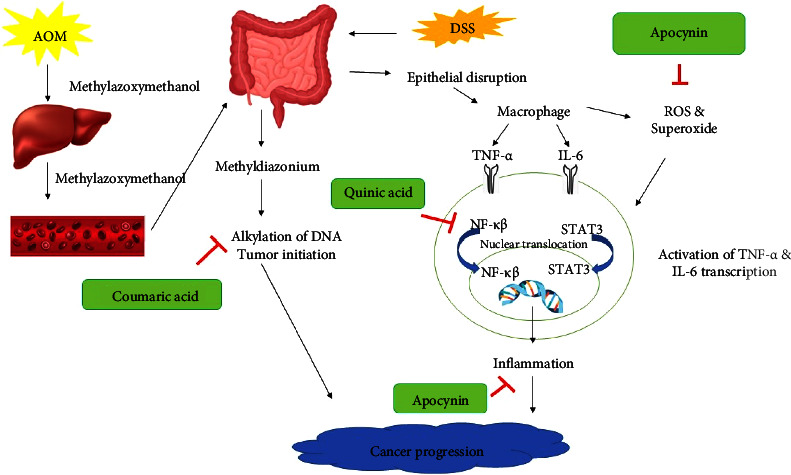
Summation diagram that illustrates the possible mechanism of MZLAE in CAC. AOM: azoxymethane; DSS: dextran sodium sulphate; TNF-*α*: tumour necrosis factor-alpha; IL-6: interleukin 6; DNA: deoxyribonucleic acid; ROS: reactive oxygen species; NF-*κβ*: nuclear factor kappa beta; STAT3: signal transducer and activator of transcription 3.

Key factors contributing to CAC include the induction of proinflammatory cytokines such as TNF-*α* and IL-6, which can promote ROS production. TNF-*α* activates the NF-*κβ* pathway, while IL-6 activates STAT3 [50]. Studies have shown that blocking NF- *κβ* signalling can reduce colon inflammation and carcinogenesis in the AOM/DSS model. IL-6 is also involved in the progression of acute inflammation to chronic colitis [51] and is upregulated in inflammation-related colon carcinogenesis. Inhibition of interleukin has been associated with protective effects against severe diarrhoea [52]. A recent study revealed that MZLAE contains several bioactive compounds, including m-coumaric, quinic acid, C16 sphinganine, 6′-O-caffeate isoorientin, and apocynin, which were detected using liquid chromatography quadrupole time of flight mass spectrometry (LC QToF MS) [8]^.^ Coumaric has been reported to possess antioxidant and anti-inflammatory properties, inhibiting the proliferation of various cancer cells [53] and promoting apoptosis [54]. Furthermore, quinic acid and apocynin demonstrate anti-inflammatory activity against colon inflammation [55, 56], further supporting the potential of MZLAE to improve oxidative stress, inflammation, and its associated carcinogenesis in the colon.  Figure 7 illustrates the possible mechanism of MZLAE on CAC based on phytoconstituents previously identified.

The anti-inflammatory properties of the extract can vary depending on the concentration used, making the dose of MZLAE critical to its effects. Higher doses may enhance the impact by more strongly regulating inflammatory pathways, whereas lower doses may initiate only a baseline anti-inflammatory response. Evaluating different doses allows for a thorough assessment of the dose-response relationship and helps identify the optimal concentration for the maximal anti-inflammatory effects. This approach helps to determine the optimal dosage to reduce oxidative stress, decrease inflammatory mediators, and moderate the overall inflammatory process. Understanding how varying doses of MZLAE affect its anti-inflammatory characteristics, scientists can better define its therapeutic potential and establish standards for its effective application in medical settings.

Current treatments for CAC such as NSAIDs and biologics can be effective but often come with significant side effects such as nephritis and high costs. MZLAE presents a potential alternative with anti-inflammatory properties that could mitigate these adverse effects, offering improvements in reducing inflammation without side effects associated with conventional treatments. However, challenges remain in translating these findings from animal models to human clinical use.

This study had limitations, particularly in the *in vivo* study, where a positive control group (CAC-induced group given a standard drug) was absent. This group was not included because there is no specific drug to treat CAC; The same chemical drugs used to treat ulcerative colitis or colon cancer are currently employed.

Further research is needed to fully understand the mechanisms underlying the beneficial effects of MZLAE. This could involve using a mouse model that more accurately represents CAC. The AOM/DSS mouse model of CAC used in this investigation, although useful, may not fully reflect the pathology of human disease, as noted in the review by Zhou et al. [5]. Therefore, it is essential to explore MZLAE's therapeutic potential in clinical settings after validating its efficacy in a more representative animal model.

## 5. Conclusions

The results of this study suggest that MZLAE may have a preventive effect on tumour initiation by reducing dysplasia development, as well as decreasing ROS, superoxide production, and inflammation through the downregulation of TNF-*α* and IL-6 proteins. These findings highlight the potential of MZLAE as a natural source of antioxidants and anti-inflammatory agents, particularly in the prevention or treatment of diseases such as colorectal cancer. Further research is necessary to fully elucidate the mechanisms underlying the beneficial effects of MZLAE and to explore its therapeutic potential in clinical settings.

## Figures and Tables

**Table 1:** DAI scoring criteria (Murthy et al., [24]).

Score	Weight loss (%)	Stool consistency	Blood in stool
0	None	Normal	Absence
1	1–5	Loose stool	Absence
2	5–10	Loose stool	Presence
3	10–15	Diarrhoea	Presence
4	>15	Diarrhoea	Gross bleeding

**Table 2:** Colon length, weight, and weight-to-length ratio of mice.

Group	Colon length (cm)	Colon weight (g)	Colon weight-to length ratio (mg/cm)
Normal	8.63 ± 0.92^a^	0.35 ± 0.03^a^	40.69 ± 2.92^a^
AOM/DSS	6.56 ± 0.16^b^	0.36 ± 0.03^a^	54.10 ± 3.18^a^
AOM/DSS ± 050 mg/kg MZLAE	7.75 ± 0.53^a^	0.36 ± 0.02^a^	46.45 ± 2.81^a^
AOM/DSS ± 0100 mg/kg MZLAE	8.00 ± 0.60^a^	0.35 ± 0.03^a^	44.04 ± 4.38^a^
AOM/DSS ± 0200 mg/kg MZLAE	8.19 ± 0.97^a^	0.33 ± 0.03^a^	40.61 ± 3.98^a^

Data are expressed as mean ± SEM (*n* = 6). ^a^Values with different letters are significant at *p*  < 0.05 between groups (rows). AOM, azoxymethane; DSS, dextran sodium sulphate; MZLAE, *Manilkara zapota* leaf aqueous extract.

**Table 3:** Histological dysplasia scoring.

Group	Low-grade dysplasia score	High-grade dysplasia score
Normal	0^a^	0^a^
AOM/DSS	2.00 ± 0.22^b^	2.67 ± 0.77^b^
AOM/DSS ± 050 mg/kg MZLAE	1.33 ± 0.22^b^	1.78 ± 0.44 ^ab^
AOM/DSS ± 100 mg/kg MZLAE	0.67 ± 0.38 ^ab^	1.33 ± 0.00 ^ab^
AOM/DSS ± 200 mg/kg MZLAE	0.67 ± 0.23^a^	0.89 ± 0.44 ^ab^

Data were expressed as median ± SEM (*n* = 3). ^a^Values with different superscript letters in the same column indicate significant differences at *p* < 0.05 using the nonparametric Kruskal–Wallis test. AOM, azoxymethane; DSS, dextran sodium sulphate; MZLAE, *Manilkara zapota* leaf aqueous extract.

**Table 4:** Histological score of inflammation of the mice colonic mucosa after MZLAE administration.

Group	Scoring			
	Inflammation	The thickness of inflammatory involvement	The severity of epithelial damage	Extend of lesions
Control	0.00 ± 0.00^a^	0.00 ± 0.00^a^	0.00 ± 0.00^a^	0.00 ± 0.00^a^
AOM/DSS	2.83 ± 0.17^b^	2.17 ± 0.17^b^	2.33 ± 0.21^b^	2.67 ± 0.21^b^
AOM/DSS ± 50 mg/kg MZLAE	1.83 ± 0.17^c^	1.67 ± 0.21^b^	1.33 ± 0.21^c^	1.50 ± 0.22^c^
AOM/DSS ± 100 mg/kg MZLAE	1.67 ± 0.17^d^	1.17 ± 0.17^b^	1.00 ± 0.00^c^	1.17 ± 0.31^c^
AOM/DSS ± 200 mg/kg MZLAE	1.00 ± 0.00^d^	1.00 ± 0.00^c^	0.83 ± 0.17^c^	0.83 ± 0.31^c^

Data are expressed as mean ± SEM (*n* = 3). ^a^Values with different superscript letters in the same column indicate significant differences at *p* < 0.05 using a one-way ANOVA test. AOM, azoxymethane; DSS, dextran sodium sulphate; MZLAE, *Manilkara zapota* leaf aqueous extract.

## Data Availability

All data used are included within the article.
